# A Combined Approach for Health Assessment in Adolescent Endurance Runners

**DOI:** 10.3390/healthcare9020163

**Published:** 2021-02-03

**Authors:** Tomas K. Tong, Julien S. Baker, Fiona L. Henriquez, Qingde Shi, Haifeng Zhang, Zhaowei Kong, Jinlei Nie

**Affiliations:** 1 Department of Sport, Physical Education and Health, Hong Kong Baptist University, Hong Kong 999077, China; tongkk@hkbu.edu.hk (T.K.T.); jsbaker@hkbu.edu.hk (J.S.B.); 2 Institute of Biomedical and Environmental Health, School of Science and Sport, University of the West of Scotland, Paisley PA1 2BE, UK; Fiona.Henriquez@uws.ac.uk; 3 School of Health Sciences and Sports, Macao Polytechnic Institute, Rua de Luis Gonzaga Gomes, Macao 999078, China; qdshi@ipm.edu.mo; 4 College of Physical Education, Hebei Normal University, Shijiazhuang 050024, China; zhanghaifeng@hebtu.edu.cn; 5 Faculty of Education, University of Macau, Macao 999078, China; zwkong@um.edu.mo

**Keywords:** cardiac function, albumin, immunoglobulin, cortisol, testosterone

## Abstract

Background: It has been shown that prolonged exhaustive exercise, such as half-marathon running, could lead to transient post-exercise elevation of cardiac troponins, increase in oxidative stress, and mild decline in renal function in adolescent athletes. With increases in sports participation involving young people, there has been much interest in pre and post health evaluations following exercise. Evaluations can be used to identify pre-existing health confounders and to examine any detrimental responses that may occur post exercise. Study purpose & Methods: The purpose of this study was to evaluate pre and post exercise measures of cardiac function, serum albumin, systemic immunoglobulin (Serum IgA and IgG), cortisol and testosterone in adolescent (age: 16.2 ± 0.6) male endurance runners performing in 21-km maximal run. Results: Results revealed that cortisol, IgA and IgG levels significantly decreased 2, 4, and 24 h post exercise compared to pre-exercise levels (*p* < 0.05). Testosterone levels reduced 4 h post exercise (*p* < 0.05) but were restored to baseline values following 24 h. There were no changes recorded for albumin levels post exercise (*p* > 0.05). ECG assessments did not show any abnormalities at the T wave axis, ST segments and Q wave pre or post exercise. Conclusions: The findings from this study suggest that a single bout of prolonged maximum running is not likely to induce abnormal electrical activity in the heart, but does decrease serum immunoglobulin, and homeostasis of anabolic and catabolic hormones in trained adolescent endurance runners.

## 1. Introduction

Recently, there has been an increase young peoples’ participation in sport training (e.g., long-distance running) [1]. Accordingly, considerable interest has emerged regarding the health benefits and pitfalls of youth involvement in sport [2]. There is significant evidence supporting the inclusion of regular physical activity as beneficial for the prevention of chronic diseases and improvements in the general health of young people [3]. However, participation by young athletes in intensive training and competition also gives rise to several concerns. For example, pre-participation cardiovascular evaluation of young competitive athletes has been recommended in order to identify cardiovascular conditions that may cause sudden death or cardiovascular injury [4]. Researchers have also revealed concerns about strenuous training regimes impeding growth and development [2]. Additionally, there is evidence suggesting that intense training in young people often causes psychological damage [5].

The half-marathon race is a popular long-distance running race for junior runners. The race covers a distance of 21 km where the runners generally run at an intensity corresponding to ~80% of their VO_2max_ [6]. Such intense metabolic demand places a considerable load on various biological systems of the runners for a period of not less than an hour. Previous studies have evaluated the health impacts of intensive running in adolescent athletes and found that the antioxidant defence capacity of the runners did not counteract exercise-induced oxidative stress completely, after an exhaustive 21-km run [7]. Moreover, 21-km running-induced a mild decline in renal function in the runners. This was in contrast to the rapid normalization in adult athletes and persisted for up to 24 h post-workout [8]. Besides, a transient post-exercise elevation of cardiac troponins above myocardial injury cutoff points appeared in the runners after the exhaustive 21-km run. The time course of the release of the myocardial biomarkers were not the same as those observed in clinical situations of myocardial infraction [9]. The transient post-exercise elevations of cardiac troponins was further found to be gender-specific, with higher levels of cardiac troponins appearing in young males than in training-background- and sexual-maturity-matched young female runners [10].

In order to effectively understand the responses of young athletes to intense training and competition, it is essential to further recognize potential risks and identify means of circumventing underlying health problems. Therefore, the purpose of this study was to investigate any cardiovascular abnormalities of young male endurance runners at rest and following an exhaustive 21-km run using an electrocardiogram. A further aim was to examine the serum concentrations, pre- and post-exercise, of immunoglobulin A (IgA) and immunoglobulin G (IgG); as well as the hormonal steroids testosterone and cortisol. These markers have been commonly used to assess and monitor immune function and stress levels in different athletic groups [11] while providing additional information on inflammatory indices. Elevated immunoglobulin levels are an indicator of infection, and elevated levels of cortisol can suppress immune responses. These are potential issues of major concern, especially for athletes performing under stressful conditions, that include high-intensity exhaustive exercise [12]. In addition, testosterone can down regulate inflammation, including the reduction of lymphocytic activities and the synthesis of proinflammatory mediators [13]. Additionally, low levels of this hormone has been implicated in cardiovascular disease [14].

## 2. Methods

### 2.1. Subjects

Twelve male adolescent runners aged 15–17 years with a varied number of training years and performances in half-marathon and marathon races were recruited for the study (Table 1). All subjects were trained in a local running club for long-distance runners, who had been involved in competitive events at national level. The training volume of the subjects was 60–80 km wk^−1^ and ranged between 7–21 km day^−1^. Prior to data collection subjects reported no change in dietary habits and all reported consuming a general healthy diet. After receiving exhaustive background information relating to the purposes and constraints of the study, and the potential benefits and risks involved in the exercise tests and measurements, subjects and their guardians gave their written informed consent for participation in this experiment. The study design and protocol were approved by the Macao Polytechnic Institute Ethical Committee on the Use of Human & Animal Subjects (No. RP/ESCSD-04/2020).

### 2.2. Protocols

Anthropometric measurements and assessment of aerobic capacity was carried out during the first laboratory visit. Body composition was measured by bioelectrical impedance analysis using the InBody 3.0 body composition analyser (Biospace Co. Seoul, Korea). A week later, during the subsequent laboratory visit, all athletes were required to perform a 21-km run using an ‘‘all-out’’ effort. To minimize the negative effects of boredom of prolonged running all subjects performed the running test together in one trial on a standard 400-m track at an ambient temperature of 5 °C and a relative humidity of ~35%. As a pre-post intervention study, the running trial was conducted during the pre-season training period and started at 08:00 am. Subjects were requested to refrain from intense physical exercise 24 h prior to testing. The diet of each subject in the team, as well as their food intake during the experimental period of >24 h were under the close supervision of the team dietitian.

Before the 21-km run, a 12-lead electrocardiogram (ECG) was recorded on all subjects to investigate any potential cardiovascular abnormalities. Thereafter, a general warm-up including sub-maximal running was performed. Pre-exercise (Pre-ex) venous blood samples for biochemical analyses were taken immediately prior to the warm-up and participation in the 21-km running test. During the running test, the subjects’ running speed was self-selected. Their exercise heart rate (HR) was recorded throughout the run using a validated telemetry system (Polar S810; Polar Electro, Kempele, Finland). The HR data was recorded at 5-sec intervals throughout the running trial and was averaged for further analysis. Water replacement ad libitum took place voluntarily at every 5th kilometer (km). The distance in km covered during the test was visible to subjects following completion of each lap. After completion of the running test, the subjects’ ECGs were measured twice within four hours at two-hour intervals for preliminary examination for any cardiovascular abnormalities. Blood samples were taken at two (2-h), four (4-h) and twenty-four (24-h) hours post exercise. All samples were measured in duplicate and were analyzed for albumin, serum testosterone, cortisol, immunoglobulin A (IgA), immunoglobulin G (IgG), hematocrit and hemoglobin. Finally, all subjects could drink water ad libitum throughout the post-run recovery period, as outlined during their routine training.

### 2.3. Graded Treadmill Run for the Determination of Aerobic Performance

A graded treadmill-running test was conducted on a motor-driven treadmill (Trackmaster, TMX425, JAS Fitness Systems, Newton, Kansas, USA) in an air-conditioned laboratory at 18 °C and 40% relative humidity. After reporting to the laboratory, subjects were weighed. Prior to the test, subjects completed a standardized warm-up consisting of 5-min sub-maximal running on treadmill at self-selected running speed followed by 5-min stretching exercises. The test commenced at a speed of 8 km h^−1^. The running speed was increased using increments of 1 km hr^−1^ for each minute while the gradient was maintained constantly at 0%. The test was carried on until either the increase in oxygen uptake (VO_2_) was less than 2.1 mL kg^−1^ min^−1^ while respiratory exchange ratio ≥ 1.15 with increase in speed or volitional exhaustion. VO_2max_ was recorded as the highest 30-s average value of VO_2_ during the test.

### 2.4. ECG Measurements

Subjects were instructed to rest for 5 min in a supine position for both pre- and post-exercise ECG assessments. The MAC 1200 ST Resting ECG System (GE Medical Systems Information Technologies, Freiburg, Germany) was used for cardiac evaluation and analysis. The placement of the electrodes and the resting 12-lead ECG interpretation including the normal limit of T wave axis, ST segments and Q wave were assessed by a medical physician using guidelines provided by American College of Sports Medicine [15].

### 2.5. Blood Analysis

Five millimetres of venous blood was drawn from an antecubital vein with subjects sitting in a relaxed position at each time point pre- and post-exercise. An aliquot was obtained for the determination of whole blood hematocrit and hemoglobin. The remaining blood samples were put into glass tubes with no chemical additives to clot at room temperature. The clotted blood sample was then centrifuged at 2000× *g* for 20 min and serum was stored at −20 °C for later analyses of testosterone, cortisol, IgA, IgG, and albumin concentrations.

Radioimmunoassay (RIA) technology was employed using the GAMMA-C12 Gamma Counter (Diagnostics Products Corporation, Los Angeles CA, USA), using Active^TM^ testosterone RIA DSL-4000 kit and Active^TM^ cortisol RIA DSL-2100 kit (Diagnostic Systems Laboratories, Inc. Webster, TX, USA) for analyses of serum testosterone and cortisol, respectively. The intra and inter assay coefficients of variation for testosterone were 10% and 9%, respectively. The intra and inter assay coefficient of variation for cortisol were: intra: 8.4%; inter: 9.1%. Serum IgA and IgG were measured by enzyme linked immunosorbent assay (ELISA) using Beckman IgA and IgG kits (Beckman Instruments Inc. Galway, Ireland) with a Beckman Array 360 Analyzer (Beckman Coulter, Inc. Miami, FL, USA) according to the manufacturer’s instructions. The intra and inter assay coefficients of variation were less than 10%. Serum albumin was measured using an automatic random-access analyzer (Model 717, Boehringer-Hitachi, Mannheim, Germany). Hematocrit and hemoglobin were analyzed with a semi-automated hematology analyzer (F-820, Sysmex, Norderstedt, Germany).

### 2.6. Statistical Analyses

One-way analysis of variance (ANOVA) with repeated measures was used to examine the differences in testosterone, cortisol, IgA, IgG, albumin, hematocrit and hemoglobin across time points (Pre-ex, 2-h, 4-h and 24-h). The null hypothesis was that a 21 km run would have no influence on blood variables during the recovery period in adolescent endurance runners. Post-hoc analyses using Holm-Bonferroni were performed when the main effect was significant. Statistical significance was assumed if the null hypothesis could be rejected at the *p* < 0.05 level and all results were expressed as mean ± SDs. Data analysis was performed using the statistical software package SPSS 20.0 (IBM Corp., Armonk, NY, USA).

## 3. Results

During the exhaustive 21-km run, the group mean of the running time was 80.8 ± 0.8 min (ranging from 80.3 to 82.3 min) and the averaged exercise HR was 177.9 ± 12.3 b min^−1^.

### 3.1. ECG Outcome

**Figure 1:**
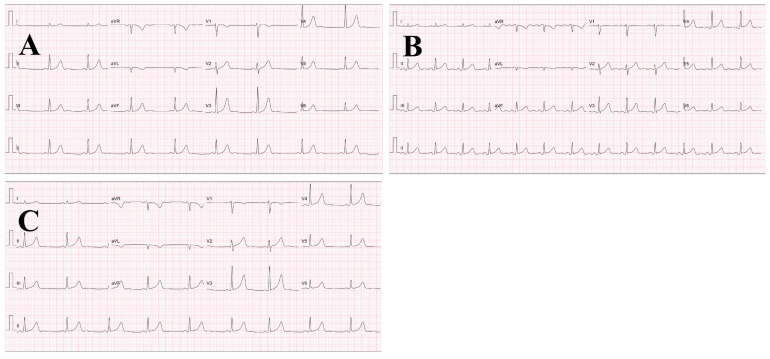
The representative pre- and post-exercise ECG strips (25 mm/s, 10 mm/mV) from a subject. (**A**) pre-exercise; (**B**) 2 h after exercise; (**C**) 4 h after exercise.

The results for pre- and post-exercise ECG assessment did not show any abnormal signs at T wave axis, ST segments and Q waves (Figure 1). The groups mean running time and exercise heart rate were 80.8 ± 0.8 min (range: 80.3–82.3 min) and 178 ± 12 b.min^−1^ (corresponding to 75.3 ± 7.1% $VO_{2}$_max_), respectively. Despite all subjects’ fluid intake at every 5th km throughout the run, there was a significant difference between pre- (59.5 ± 6.0 kg) and post-exercise (58.5 ± 5.8 kg) body weight (*p* < 0.05).

### 3.2. Blood Analysis

Levels of immunoendocrine markers changed following the running event. Post exercise cortisol levels significantly decreased at 2-h (*p* < 0.01), 4-h (*p* < 0.001) and 24-h (*p* < 0.05), when compared to pre-exercise values. In addition, IgG and IgA levels also significantly decreased post exercise compared to pre-exercise levels (2-h *p* < 0.05, 4-h and 24-h *p* < 0.05 for IgG; 2-h *p* < 0.01, 4-h and 24-h *p* < 0.05 for IgA). There was a notable decrease in testosterone at 4 h post exercise (*p* < 0.05), but levels returned to pre-exercise values after 24 h (Table 2). Conversely, haematocrit, haemoglobin, and albumin did not alter significantly from the pre-exercise values, suggesting that the changes in the immunoendocrine markers post exercise were not attributed to changes in plasma volume.

## 4. Discussion

The purpose of this study was to assess the cardiovascular, physiological and biochemical responses of male adolescent runners to an endurance running event, and to evaluate any potential risks and underlying health problems.

### 4.1. Testosterone

The results of the study indicated that there was a notable decrease in testosterone concentrations at 4 h after the exhaustive 21-km run (*p* < 0.05), but levels returned to pre-exercise values after 24 h. This is in contrast to other studies, which have determined that prolonged submaximal exercise can result in elevated circulating testosterone levels [16], or no major differences in direct comparisons of exercise testosterone responses between endurance-trained (with low testosterone levels) and untrained male controls [17]. These changes in testosterone during submaximal exercise may be due to the interaction of several factors. Initially with submaximal exercise, haemoconcentration may produce elevations in testosterone levels [18]. However, as exercise continues a reduction in testicular production and secretion of testosterone may be observed, partly due to declining testicular blood flow that may possibly result from exercise-induced sympathetic vasoconstrictor tone to the testicles [19,20]. Further, the liver and skeletal muscle cells may also play a role in reduction in testosterone levels. If the liver is not be able to totally clear the hormone, skeletal muscle cells will facilitate uptake [21]. Hormone response to exercise is influenced by training status and circulating endogenous hormone profiles are more dependent on the type of exercise or intensity rather than on exercise volume [22]. Such information could be useful in designing training regimes that promote the most favourable ratio for sports performance of anabolic and catabolic hormones.

### 4.2. Cortisol

Although true resting levels of cortisol do not differ between trained athletes and sedentary controls, exercise and recovery periods are associated with elevated cortisol release. For example, even 15 min of submaximal cycling exercise has been demonstrated to elevate post-exercise cortisol levels in saliva [23]. A study of marathoners found salivary cortisol levels elevated during the race but were maximal at 30 min following race completion [24]. In the present study, we found that at the time points of 2-h, 4-h, and 24-h post exercise, cortisol levels were significantly lower than pre-exercise values. The decrease in cortisol levels is in agreement with previous findings that submaximal treadmill running performed for 30 min in the morning (07:00) stimulated cortisol release for approximately 2 h post exercise [25]. The continuing decline of the post-exercise cortisol from the peak 2 h after the exercise followed a trend continuing to be lower than the pre-exercise levels. This was probably due to the circadian rhythm effects of cortisol release where the pre-exercise cortisol level recorded was possibly the highest resting level during the day [25,26].

Cortisol concentration is a good measure of recovery in long-distance runners [24]. Aerobic and anaerobic muscle fibres need time to repair and recover from hard workouts to improve their capacity for exercise. Cortisol can reduce the activity of muscle repair and growth and therefore its reduction is an ideal scenario for an athlete [27,28]. Studies have also established that the ratio between free Testosterone/Cortisol is important for athletic training status, since elevated cortisol decreases testosterone [29]. An elevated cortisol/testosterone ratio is an indication of overtraining, so the modulation of this ratio is important for those athletes who are susceptible to overtraining and injury [30,31].

In addition, diet has an impact on cortisol levels. Carbohydrates consumed during exercise have been shown to decrease the immune system and cortisol response within 30 min after 6 × 15 min maximal running activity, when compared to a non-caloric, sweetened placebo [32]. Athletes exercising in a carbohydrate-depleted state experience larger increases in cortisol and a greater suppression of immune function [33]. Although the food intake of each runner during the experimental period of >24 h was not recorded in the present study, it was strictly in compliance with the instructions of the team dietitian and coaches and did not vary from their regular food intake. Accordingly, an important take home message for athletes and coaches is that the regulation of blood and body glucose levels are within the athlete’s and coaches’ control and mismanagement of these variables can seriously impact on athletic health and ability to enhance exercise performance and training adaptations.

### 4.3. Immunoglobulin Levels

Antibody immunoglobulin levels, IgA, are useful indicators of immune function during exercise. It has been reported that salivary IgA decreased immediately after intense endurance exercise including swimming, distance running, cycling and rowing, whereas levels of IgG remained unchanged [34,35]. In this study, we observed a decrease in systemic IgA and IgG levels in the first 2 h post 21-km endurance run, which was maintained at the 24-h time point. The decrease in the immunoglobulin levels has been considered as a temporary response of immune suppression induced by exercise, heightening the risks of opportunistic infections and compromising performance outcomes in the days thereafter [36,37]. However, recent studies [38,39] advocated that rather than detrimental to immunological health, an acute bout of exercise could improve immune surveillance, and ultimately, enhance antibacterial and antiviral immunity. Although the present findings could not enrich markedly the current knowledge about the various underlying mechanisms for the decline of the immunoglobulin levels and its influences on the immunological health in adolescent runners, there was no clinical case of infectious disease, such as upper respiratory tract infection, reported in the runners in the present study in the days after the run. In fact, the occurrence of infectious disease in the runners, according to the team physician, was minimal all year round.

Usually, salivary IgA is assessed in exercise trials, as this method is non-invasive and easy to access. However, we deemed it necessary to study systemic immunoglobulin due to the wider scope of this study. It is widely reported that salivary IgA decreases post endurance exercise and systemic levels remain unchanged or even slightly increase [35,40]. However, this contrasts with our findings. It is generally agreed that exercise-induced changes in the immunoglobulin levels partly depend upon the intensity and the time of exercise [41]. Moderate exercise has been demonstrated to enhance the humoral immunological parameters of IgA and IgG while maximal exercise suppressed these parameters [42]. The current findings of the decrease in the serum IgA and IgG subsequent to the “all-out” 21-km run in runners are in agreement with a previous notion that while moderate and short drills in the training of long-distance runners could strengthen the immune system of the athletes, prolonged intensive exercises, such as half-marathon run, are associated with the adverse effects upon immunological health [41].

It is generally agreed that nutritional deficiencies are associated with depressed immune function and a decline in resistance to illness in athletes [43]. Both macro and micronutrients are essential in the maintenance of immune function [44]. Previous studies have demonstrated that athletes exercising in a carbohydrate-depleted state increase circulating stress hormone (e.g., adrenaline and cortisol), and result in a greater perturbation of several immune function indices [45,46]. Conversely, consumption of 30 to 60 g carbohydrate per hour as a 6.4% *w/v* beverage during prolonged exercise has been shown to attenuate certain exercise-induced immunodepressive responses [47]. In the present study, the decrease in the immunoglobulin levels post exercise in the runners had not been influenced by any particular immune supplementation before or during the exercise, or any dietary immunostimulant. The potential immune impairment following intense exercise did not result in any apparent infection in the runners and this might be attributed to their well-balanced diet that was closely supervised by the team dietitian. While there were no deviations from the normal eating habits of the runners, detailed nutritional information and dietary analysis was not recorded in this study. Recording of nutritional status should be noted in future studies. Underestimating nutrient intake could compromise the health and well-being of the athletes and contribute to a negative immune function response following exercise [44].

### 4.4. Limitations and Practical Applications

In the present study, the sample size was not based on a priori power calculation, partly due to the inconsistent confidence interval of exercise-induced alterations in the hormonal steroids and immunoglobulins from previous works. Nevertheless, the sample size (*n* = 12) in the present study was comparable to those employed in previous studies examining the changes in the same serum variables in trained individuals during exercise [18,25,29]. The food intake of runners during the experimental period was not recorded. Nutritional intake, in particular carbohydrates, influence the changes in cortisol and immune function in response to exercise and this needs further consideration in future research. This may be useful for coaches and athletes in relation to performance outcomes and training responses to different exercise intensities. Further to this, we also suggest that the effects of carbohydrate, fats and ketogenic diets are investigated in future studies in relation to cortisol and immune function responses. Poor understanding of nutrient intake could compromise the health and well-being of athletes and contribute to a negative immune function and hormonal response following exercise. Coaches and trainers should be aware of this during preparation for performance and during competition. In addition, systemic antibody production decreases may lead to upper respiratory tract and other infections that may be detrimental to athletic performance and performance preparation.

## 5. Conclusions

In this study, we observed that changes in immunoendocrine markers could be a useful indication of health status for young male endurance athletes. We note that a single bout of all-out 21-km running is not likely to induce abnormal electrical activity in the heart, but we identified a decrease in testosterone, cortisol and systemic antibody production. Since nutritional intake, in particular carbohydrates, influence a decrease in cortisol and immune function biochemistry we recommend that a detailed nutritional analysis of the participating subjects be recorded in future studies.

## Figures and Tables

**Table 1:** Subjects’ physical characteristics, training years and personal best in marathon race are shown (*n* = 12).

Variables	Mean ± SD	Range
Age (year)	16.2 ± 0.6	15.1–17.1
Body height (cm)	173.8 ± 5.2	165.5–182.2
Body weight (kg)	60.3 ± 5.7	51.6–70.2
Percentage of body fat (%)	10.9 ± 2.6	5.9–16.9
Fat free mass (kg)	50.9 ± 5.5	40.5–58.7
Training years (year)	3.2 ± 1.8	1.3–8.3
VO_2max_ (mL kg^−1^·min^−1^)	59.5 ± 5.3	50.6–68.9
Personal best in half-marathon (min)	73.9 ± 2.2	71.1–77.0
Personal best in full-marathon (min)	175.5 ± 8.0	163.7–187.3

**Table 2:** Mean ± SD of serum testosterone, cortisol, Albumin, immunoglobulin A (IgA), immunoglobulin G (IgG), haematocrit and haemoglobin of male runners aged 15–17 years (*n* = 12) immediately before (Pre-ex) and two (2-h), four (4-h), and twenty- four (24-h) hours after 21-km run are shown.

Variables	Pre-ex	2-h	4-h	24-h
Testosterone (ng dL^−1^)	410.5 ± 102.2	374.6 ± 131.9	331.3 ± 110.4 *	474.6 ± 148.6
Cortisol (μg dL^−1^)	13.3 ± 2.8	9.9 ± 2.0 **	7.8 ± 3.2 ***	8.9 ± 3.5 *
Albumin (g L^−1^)	44.5 ± 1.2	45.2 ± 1.8	44.8 ± 1.1	44.3 ± 1.4
IgA (g L^−1^)	1.77 ± 0.66	1.68 ± 0.61 **	1.70 ± 0.63 *	1.67 ± 0.63 *
IgG (g L^−1^)	10.70 ± 2.61	9.91 ± 2.30 *	9.80 ± 2.18 *	9.60 ± 2.23 **
Hematocrit (%)	43.5 ± 3.1	42.9 ± 2.8	42.2 ± 2.3	42.4 ± 2.0
Hemoglobin (g dL^−1^)	14.2 ± 0.8	13.9 ± 0.9	14.0 ± 0.8	14.1 ± 0.8

* Significantly different from corresponding Pre-ex value, * *p* < 0.05; ** *p* < 0.01; *** *p* < 0.001.

## Data Availability

The data presented in this study are available on request from the corresponding author. The data are not publicly available due to the wishes of some subjects.
